# Disease of the Tongue and Mouth

**Published:** 1842-04

**Authors:** 


### DISEASE OF THE TONGUE AND MOUTH.

Dr. James C. Harris, of Jefferson, Cherokee county, Alabama, in a letter to one of the Editors, dated January 19th, 1842, relates a very interesting case of disease of the tongue and mouth. He gives the following history of the patient:

"During the spring of 1836, Col. John Lowry, aged 64, a lawyer by profession, and then a resident of Talladiga, Alabama, had a most violent attack of bilious fever. In the course of the treatment, it was thought necessary by the physicians in attendance to administer calomel in large quantities; he accordingly took 50 or 60 grains at a dose, on several different days. Profuse salivation ensued, and recovery was slow. After this, he enjoyed a tolerable degree of health until the summer of 1840, when he was seized with an inflamed and ulcerated tongue, sore and swollen gums and lips, and a profuse flow of saliva. The fetor of the breath and other attendant symptoms resembled the effects of mercury so much, that the patient believed his former ptyalism had returned, and so expressed himself, although he had not taken a particle of mercury, in any form whatever, since his attack of fever in 1836. Being then in the midst of a heated political contest, but little if any thing was done in the way of treatment until August. At that time, in addition to the affection of the mouth and fauces, Col. L. presented the following symptoms: skin sallow, particularly that of the face; pulse slightly accelerated; centre and base of the tongue covered with white fur; nausea, and slight derangement of the bowels, most generally of a dysenteric character, but occasionally the reverse. I supposed the condition of the tongue and mouth to depend upon a depraved state of the liver and chylopoietic organs, and accordingly prescribed a mercurial cathartic, which acted well, but excited an unusual and almost intolerable degree of nausea, that did not subside for several days. It was then repeated with nearly the same consequences, the nausea being if any thing augmented; the flow of saliva was also increased, and the intumescence of the tongue and adjacent glands was observed to be greater. Sulphur was now given in tea-spoonful doses, and a detergent wash applied to the mouth and throat. The local inflammation subsided to its former condition, at which it remained stationary for several months, when it gradually became worse, and again excited serious attention. The derangement of the bowels continued during all this period, having undergone no amelioration from the remedies employed.

I now directed a pill, containing two grains of calomel and one-fourth grain of opium, to be taken every night at bed-time. The first pill had the effect of lessening the number of alvine discharges; after the second, the bowels were still further improved, but the condition of the mouth was thought to be aggravated. Nevertheless a third was administered, which was followed by complete mercurialization. Recourse was again had to the sulphur and the detergent gargle previously mentioned, but with no better success than in the first instance, the condition of the mouth remaining much the same. Col. L.'s health was now so much impaired by the constant discharge of saliva, the nausea and loss of appetite, and the accompanying diarrhœa, as to prevent him almost entirely from attending to business. Subsequently, the intestinal derangement and the general health were somewhat improved by the use of the tinct. ferri muriat., but in other respects he remained the same. For the last few weeks the patient has been using the elixir vitriol, with decoction of sarsaparilla *ad libitum*, but without any apparent benefit."

Dr. Harris asks, in conclusion, if it is possible that his patient is laboring under ptyalism from mercury *taken four years previously?* if not, what form of disease must it be considered, and on what does it depend?

